# Japanese attitudes toward cell donation in human brain organoid research: many oppose broad consent

**DOI:** 10.3389/fgene.2025.1606923

**Published:** 2025-08-22

**Authors:** Masanori Kataoka, Mayu Koike, Tsutomu Sawai

**Affiliations:** ^1^ Uehiro Division for Applied Ethics, Graduate School of Humanities and Social Sciences, Hiroshima University, Higashi-Hiroshima, Japan; ^2^ School of Engineering, Institute of Science Tokyo, Tokyo, Japan; ^3^ Graduate School of Humanities and Social Sciences, Hiroshima University, Higashi-Hiroshima, Japan; ^4^ Centre for Biomedical Ethics, Yong Loo Lin School of Medicine, National University of Singapore, Singapore, Singapore

**Keywords:** bioethics, organoids, informed consent, personal autonomy, public opinion

## Abstract

**Introduction:**

Under broad consent, donors are not informed about the specific research projects using their cells; this may lead to the use of cells in ways that conflict with donors' moral beliefs. In recent years, this issue has been raised in human brain organoid research. However, previous studies on the public’s attitude toward human brain organoid research have either overlooked cell donation or consisted of small-scale qualitative studies.

**Methods:**

We conducted an online survey on Japanese citizens' attitudes toward cell donation for human brain organoid research, gathering 326 responses.

**Results:**

When informed that donated cells could generate human brain organoids, 36% of participants disapproved of broad consent, while 37% said their stance depended on the case. Reasons for opposition included the need for study-specific explanations, autonomous decision-making, emotional discomfort, research purpose, researchers' and institutional trustworthiness, potential misuse, and risks and benefits to participants.

**Discussion:**

Although several limitations exist, these findings may suggest that project-specific consent would be more ethically appropriate at the current stage of human brain organoid research. Since some public concerns stem from limited knowledge or misinformation, science communication could help change this situation.

## 1 Introduction

The increasing complexity and globalization of biomedical research have underscored the significance of biobanks, which collect and distribute biological materials on a large scale. Given the vast number of participants and the potential for donated samples to be used in diverse research projects over extended periods, obtaining specific consent from donors for each study presents practical challenges. One proposed solution is broad consent, wherein donors provide consent at the outset for future wide-ranging use of their biological materials.^1^ Although ethical concerns regarding broad consent have been raised and alternative approaches, such as dynamic consent, have been proposed (Master et al., 2012; Chalmers et al., 2016), broad consent remains widely adopted by many institutions.

One of the most significant concerns regarding broad consent is the potential use of donated materials for research which is morally controversial or ethically sensitive among (potential) donors (Grady et al., 2015). Such a “controversial use” involves a serious violation of donor autonomy, as donors might withhold consent if they were aware in advance that the research conflicted with their moral views (Greely, 2020).

A recent example of a possible controversial use is research involving human brain organoids (HBOs) (Boers and Bredenoord, 2018; Farahany et al., 2018; Greely, 2020; Kataoka et al., 2024a; Chneiweiss et al., 2024). HBOs are three-dimensional tissues derived from human stem cells, which develop through self-organization and obtain structural and functional characteristics similar to those of human brain tissues (Lancaster et al., 2013). As such, the production of HBOs necessitates the donation of human cells for their creation.^2^ These cells are often obtained from biobanks, and thus the consent procedure for cell donation has been re-examined recently (Lewis and Holm, 2022). Other ethical, legal or social concerns have been raised regarding the research and application of HBOs, including potential consciousness of HBOs, appropriate forms of commercialization, and inaccurate representations of HBOs (Kataoka et al., 2025b). Prospective donors may share or influenced by these concerns, perceiving this research as including controversial use of cells.^3^ Importantly, obtaining appropriate consent for HBO research is one of the most pressing ethical challenges associated with this field.

Several empirical studies have examined public attitudes toward HBOs, consistently demonstrating that HBOs can hold varying degrees of moral salience for citizens (Haselager et al., 2020; Bollinger et al., 2021; MacDuffie et al., 2023; Ravn et al., 2023; Evans, 2024; Ota et al., 2024; Villanueva et al., 2025). However, it remains unclear whether these concerns are significant enough to cast doubt on broad consent, specifically, whether HBO research truly constitutes a controversial use of donated cells.

To the best of our knowledge, there have been four studies that have directly explored perspectives on cell donation or preferred consent models so far. Haselager et al. (2020) conducted semi-structured interviews on HBO research with 28 people in the Netherlands. Participants generally showed high motivation to donate cells for HBO research, but also emphasized the importance of explanation of the purpose of the research. Bollinger et al. (2021) surveyed 60 citizens in the United States regarding organoid technology, including HBOs. Overall, there was a demand for transparent consent that reflects autonomy, and particular caution was noted regarding HBO research. MacDuffie et al. (2023), which conducted semi-structured interviews on informed consent in HBO research with 67 US citizens, is most interesting for us. Participants generally supported broad consent, particularly for research related to health. However, ongoing communication was also considered important: participants wanted to know the progress and results of the research, as well as notification or an opportunity to make a new decision regarding research that exceeds the scope of their initial consent. Finally, Ravn et al. (2023) reported the results of a series of workshops on organoid technology (including HBOs) held in three counties (Italy, Greece, and Denmark) with a total of 51 participants. One of the important findings is that most participants opposed the unrestricted use of donated cells in organoid technology, and preferred to impose some restrictions on the scope of use at the time of consent or to re-obtain consent ongoingly.

These studies provide valuable deep insights of participants. However, as all of them have been relatively small-scale qualitative investigations, they may not be the most relevant to the current question. For, determining whether a particular use of a sample is controversial depends on the distribution of ethical perspectives within a relevant population. Unfortunately, quantitative surveys conducted thus far have not directly addressed issues of cell donation and consent (Evans, 2024; Ota et al., 2024; Villanueva et al., 2025). To address this gap in the literature and examine the possibility of the controversial use of donated cells in HBO research more directly, we conducted a social survey among Japanese citizens.

## 2 Materials and methods

### 2.1 Participants and recruitment

We conducted an online survey, *Public Survey on Brain Organoid Research* in Japanese, on 8 December 2022. Participants were recruited through Lancers, Inc., a Japanese crowdsourcing service, and we obtained 326 valid responses from a total of 353 participants).^4^ Participants received 500 Japanese yen as compensation for their participation.^5^ The main demographic characteristics of the participants are summarized in Table 1. The median completion time for the entire questionnaire was approximately 19.7 min (SD = 13.9).^6^ For details on recruitment and further demographic information, see the Supplementary Material.

**TABLE 1:** Brief demographic information of the participants. For more detail, see the Supplementary Material.

Brief demographic information	N
*Total*	326
*Country*	
Japan	326
*Age (in years)*	
≤19	1
20–29	23
30–39	99
40–49	138
50–59	50
60–69	15
≥70	0
*Gender*	
Female	126
Male	200
Other	0
Declined to state	0
*Education*	
Elementary School	0
Junior High School	7
High School or Technical College	66
Vocational School	36
Junior College	23
Bachelor’s Degree	179
Master’s Degree	13
Ph.D	2
Other	0

### 2.2 Procedure

#### 2.2.1 Structure of the questionnaire

After obtaining written informed consent, participants were presented with a comprehensive questionnaire. The questionnaire consisted of the following distinct sections:

1. Attention check
2. Brief overview of brain organoid research
3. Comprehension test
4. Awareness-related question regarding brain organoids
5. Questions about *in vitro* brain organoid research
6. Questions about the transplantation of HBOs into animals
7. Questions about the transplantation of HBOs into humans
8. Questions about cell donation for brain organoid research
9. Demographic questions

Since each section was designed to address different research questions, we determined that publishing the survey results across multiple publications would be appropriate. In this paper, we examined the results from Sections 4 and 8, which are particularly relevant to cell donation for HBO research. The analysis of responses from Section 5 has already been published in Sawai et al. (2025), where the same participants and, in part, the same procedures as in this study were described. Below, we provide a detailed explanation of the sections relevant to this paper (for further details of the questionnaire, see the Supplementary Material).

#### 2.2.2 Brief overview of brain organoid research

Since HBO research is novel and complex, we provided a brief overview of the study at the beginning of the questionnaire to ensure that participants were at least minimally informed. This section introduced the following key points, accompanied by images:

• What are brain organoids?
• Research purposes
• Ethical considerations
• Present and future trajectory of brain organoid research

#### 2.2.3 Awareness-related question regarding brain organoids

In this section, we asked participants, “Before participating in this survey, were you aware of brain organoids?” Participants selected from the following options: “I knew enough about brain organoids to be able to explain them to some extent” (*well-known*); “I had heard of brain organoids, but did not know much about them” (*known*), and “I did not know about brain organoids” (*unknown*).

#### 2.2.4 Questions about cell donation for brain organoid research

##### 2.2.4.1 Explanation

This section began with a brief explanation of cell donation for HBO research. To ensure that cell donation was conceptually relevant to all participants, we focused on hiPSCs and provided the following explanation:

“Human iPSCs are one of the cell types needed to create HBOs. iPSCs are primarily derived from blood cells, meaning that blood donation is required to generate HBOs. Cell donors are typically provided with a detailed explanation of the research and its associated risks. Only after fully understanding these details do they decide whether to consent to donating their cells for that research.”

Following this explanation, participants proceeded to the following three questions. As this initial description introduces project-specific consent as the standard consent model, it can be reasonably inferred that participants considered this model while responding to the subsequent questions, with the exception of the final question, which explicitly addressed broad consent.

##### 2.2.4.2 Willingness

Participants were first asked, “Would you be willing to donate your cells to create iPSCs for human brain organoid research?” Answers were collected after clarifying that there was no request for cell donation later on in the questionnaire.

For this question, participants selected from the following response options: “I would be willing to donate my cells” (*willing to donate*); “It would depend on the purpose of the study” (*it depends*); and “I would not be willing to donate my cells” (*not willing to donate*).

Importantly, due to the structure of the questionnaire, participants had the opportunity to consider various aspects of HBO research before responding to this question. These included the potential contributions of HBO research to basic and medical science, ethical concerns, transplantation into animals, and the possible future transplantation of brain organoids into humans. Therefore, it can be reasonably assumed that participants considered both the advantages and disadvantages of HBO research when answering this and subsequent questions.

##### 2.2.4.3 Purposes

To determine which research purposes participants particularly supported, we asked, “For which of the following purposes would you be willing to donate your cells?” The following seven research purposes were presented in a random order, and participants were allowed to select multiple answers: “To deepen our understanding of the brain (for example, brain development, structure, and function)” (*basic research*); “To elucidate the causes of brain-related diseases” (*disease exploration*); “To develop treatments for brain-related diseases” (*treatment development*); “To develop drugs for brain-related diseases” (*drug discovery*); “To elucidate the causes of brain-related diseases affecting you, or to develop treatments and drugs for them” (*medical applications for themselves*); “To elucidate the causes of brain-related diseases in your family, or to develop treatments and drugs for them” (*medical applications for family*); and “I would not be willing to donate my cells for any of the purposes above” (*nothing*).

Additionally, we provided a text box where participants could describe other purposes for which they would not be willing to donate their cells, if applicable.

##### 2.2.4.4 Broad consent

Before responding to the question, participants were provided with the following brief description of broad consent. Given the purpose of this study, the description emphasized the possibility that donated cells could be used to create HBOs without the donor’s knowledge:

“At some research institutions, researchers have the authority to determine how donated cells will be used. Therefore, if you were to donate your cells to such an institution, brain organoids could be produced from your donated cells.”

Participants were then asked, “Would you be willing to donate your cells to such an institution?” They selected from the following response options: “I would be willing to donate my cells” (*willing to donate*); “I would not be willing to donate my cells” (*not willing to donate*); “I do not know” (*do not know*); and “It depends” (*it depends*). Additionally, participants were given the opportunity to describe the reasons behind their responses.

## 3 Results

### 3.1 Awareness

Most participants (n = 297, 91%) were unaware of HBOs prior to this study. A smaller proportion of participants had some prior knowledge of HBOs, with 7% (n = 24) reporting that they had heard of them and 2% (n = 5) indicating that they were familiar enough to explain them to some extent. Collectively, those who had prior knowledge of HBOs accounted for less than 10% of the participants.

### 3.2 Willingness

**FIGURE 1:**
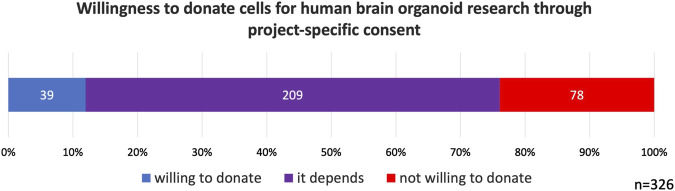
Willingness to donate cells for human brain organoid research through project-specific consent. 64% of participants indicated that their willingness to donate cells would depend on the circumstances.

Regarding willingness to donate cells for HBO research, 12% of participants indicated that they would be willing to donate. The majority of participants (64%) stated that their willingness would depend on the specific details of the study, while 78 participants (24%) reported that they would not be willing to donate (Figure 1). As previously noted, it can be reasonably inferred that participants considered project-specific consent when responding to this question.

### 3.3 Purposes

**FIGURE 2:**
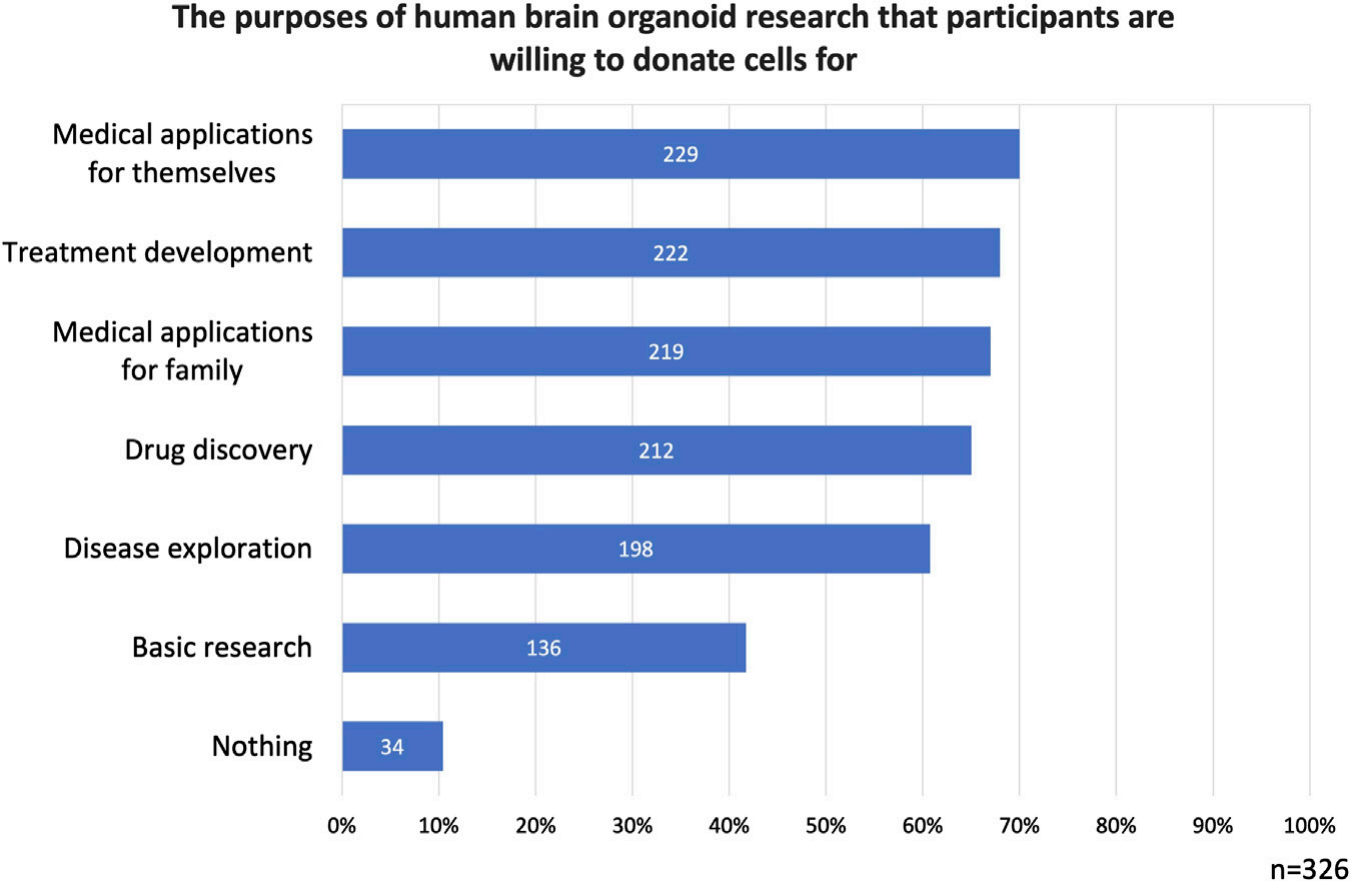
The purposes of human brain organoid research that participants are willing to donate cells for. Participants expressed greater support for donating cells for medical research purposes compared to basic research.

Regarding research purposes for which cells could be donated, *basic research* was selected by 42% of the participants. *Disease exploration* was chosen by 61% of the participants, *Treatment development* by 68%, and *drug discovery* by 65%. *Medical applications for themselves* were selected by 70% of the participants, and *medical applications for family* was chosen by 67% of the participants. Finally, *nothing* received 34 selections (10%) (Figure 2).

Logically, all 78 respondents who previously indicated *not willing to donate* should have selected *nothing* in this question. However, only 34 participants did so. This discrepancy may suggest that individuals who were initially reluctant to donate cells when asked in a general context reconsidered their stance when the purpose of donation was specified.

### 3.4 Broad consent

**FIGURE 3:**
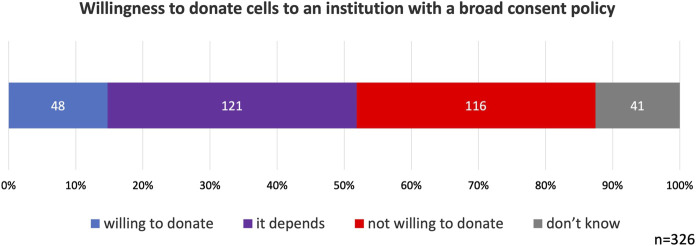
Willingness to donate cells to an institution with a broad consent policy. Participants were informed that donated cells could potentially be used in human brain organoid research. 36% of participants did not support broad consent.

Overall, 15% of the participants indicated that they would be willing to donate their cells to institutions adopting broad consent. 37% of the participants responded with *it depends*. Notably, 36% of participants selected *not willing to donate*. *Don’t know* was selected by 13% of participants (Figure 3).

To better understand the conditions under which broad consent would be supported, we examined the reasons provided by participants who selected *it depends.* Among the 121 respondents who chose this option, 64 participants (53%) provided an explanation for their choice. After one of the authors coded the responses, we identified the following eight categories of reasons (with overlapping responses):

• Explanation: Participants wanted to be informed about the nature and purpose of the research (n = 19, 30%).
• Trust: Participants required trust in the researcher or the research institution (n = 13, 20%).
• Purpose: Participants wanted their donated cells to be used only for specific research purposes, primarily medical research (n = 10, 16%).
• Benefit: Participants expected that cell donation and subsequent research would provide benefits to themselves or their relatives (n = 9, 14%).
• Misuse: Participants were concerned about the unethical use of donated cells (n = 8, 13%).
• Risk: Participants were concerned about the potential risks to themselves associated with cell donation and research (n = 7, 11%).
• Others: Various reasons that did not fit into the above categories (n = 5, 8%)

Additionally, to investigate potential reasons for the *not willing to donate* responses, we analyzed the reasons given for this selection using the same approach. Among the 116 participants who chose this option, 74 (64%) provided an explanation. This analysis revealed seven categories of reasons (with overlapping responses):

• Explanation: Participants wanted to be informed about the nature and purpose of the research (n = 34, 53%).
• Negative feeling: Participants expressed discomfort with not knowing how their donated cells would be used (n = 22, 34%).
• Autonomy: Participants did not want to leave decisions about the use of their cells entirely up to the researchers (n = 19, 30%).
• Misuse: Participants were concerned about the unethical use of donated cells (n = 14, 22%).
• Mistrust: Participants expressed distrust toward the researcher or the research institution (n = 7, 11%).
• Risk: Participants were concerned about potential risks to themselves associated with cell donation and research (n = 4, 6%).
• Others: Various reasons that did not fit into the above categories (n = 8, 13%)

## 4 Discussion

### 4.1 HBO research may involve a controversial use of the donated materials

We conducted a survey on the attitudes of Japanese citizens toward cell donation for HBO research to determine whether this research involves a controversial use of biological materials by examining participants’ views on project-specific and broad consent.

First, when asked about their willingness to donate cells for HBO research under project-specific consent, 64% of participants selected *it depends*, while only 12% expressed full willingness to donate. Regarding research purposes, there was a clear difference in participant attitudes toward *basic research* (42%) compared to medical applications (65%–70%). In other words, even when provided with sufficient information about a specific study in advance, at least 24% of participants remained unwilling to donate cells for HBO research, and depending on the circumstances, especially when the purpose is basic research, the reluctance rate rises even further. This trend aligns with findings from previous studies. MacDuffie et al. (2023) reported that interviewees often stated that their willingness to consent to HBO research was contingent on the study having a medical purpose.^7^ Furthermore, Haselager et al. (2020) identified strong altruistic motivations for cell donation in HBO research. Given that, our findings suggest that participants may not fully understand the intrinsic connection between basic research and medical applications which help people more directly.^8^ This highlights the need for scientific communication strategies emphasizing the essential relationship between basic research and medical applications (Akatsuka et al., 2023).

Most importantly for our study, 36% of participants disagreed with broad consent when informed about the possibility of HBO research, representing a substantial proportion^9^. The analysis of free-response descriptions suggests that a key factor in this opposition is the lack of autonomous decision-making based on sufficient research information. This aligns with previous studies, where participants emphasized the need for clear explanations and autonomy in cell donation for HBO research (Haselager et al., 2020; Bollinger et al., 2021; Ravn et al., 2023).^10^ Beyond the need for explanation and autonomy, our results indicate that participants also prioritized the trustworthiness of researchers, concerns about unethical misuse, and the perceived benefits and risks associated with participation in HBO research. While all these factors have been identified in prior studies (Haselager et al., 2020; Bollinger et al., 2021; MacDuffie et al., 2023; Ravn et al., 2023), our data further highlights that their significance is substantial enough to drive 36% of participants to refuse broad consent.

Similarly, a comparable proportion of participants (37%) stated that their acceptance of broad consent would depend on specific circumstances. The key considerations for this group mostly mirrored those outlined above, including the explanation of the research, research objectives (with *basic research* receiving less support), trust in researchers, ethical concerns, and perceived risks and benefits. Depending on how these elements are addressed in HBO research, some among the 37% may ultimately reject broad consent.

Summarily, at least 36% of our participants do not support broad consent when donated cells may be used for HBO research, and this percentage could increase further depending on the situation. Although our survey is not perfectly representative and has some limitations (see below), the results may suggest that HBO research in Japan would involve the controversial use of donated cells.

The notably negative attitudes toward broad consent in the context of HBO research align with findings from deliberative workshops on organoid research reported by Ravn et al. (2023), where only seven out of 51 participants supported broad consent without restrictions. However, these findings contrast sharply with those from interviews conducted by MacDuffie et al. (2023), where all 67 interviewees supported broad consent if the HBO research served medical purposes. Several factors may explain this contrast. In MacDuffie et al. (2023), interviewees appeared to develop a deep understanding of the practical value of broad consent during the interviews. Additionally, unlike our study, all interviewees had prior experience with cell donation for HBO research or had acted as legal guardians for individuals who had donated. This prior exposure may have naturally predisposed them to view the possibility of their donated cells being widely used in other HBO studies more favorably. Furthermore, if regional and cultural differences between the participants (Japanese vs predominantly US citizens) play a significant role, this would suggest that the optimal approach to cell donation for HBO research varies across regions. Since the donation process should respect individuals' moral views, and moral perspectives are shaped in part by cultural factors, the ideal consent model would reflect its cultural contexts.

### 4.2 Preferable consent models

To further examine the possible implications of our survey, let us put aside the limitations of our survey tentatively and assume that HBO research in Japan *does* involve the controversial use of donated cells. How should biobanks, research institutions, and scientists respond to this situation? One option is to maintain the *status quo*. In our results, only 15% of respondents supported broad consent, and even when including those who selected *it depends*, the overall support reached just 52%. However, this is not entirely unexpected. Numerous social surveys conducted in various countries have assessed public preferences for consent models in biobanks, revealing considerable variation in support for broad consent. Master et al. (2012) reported that support rates across different countries range approximately 35%–80%. Similarly, a recent survey conducted in Japan found that only 23.9% of respondents preferred broad consent (Oikawa et al., 2023).^11^ Despite its relatively low support in some populations, broad consent continues to be widely adopted and is often considered ethically justifiable. Therefore, it could be argued that opposition to HBO research does not introduce fundamentally new ethical concerns beyond those already associated with broad consent in biobanking.

However, while maintaining broad consent may be a practical approach, it is not necessarily the most ethical option *when we know that controversial use is involved*. In fact, some scholars have proposed alternative models for cell donation in HBO research. For instance, Boers and Bredenoord (2018) advocate for a “consent for governance” model in organoid research, where donors provide consent for the governance of research including privacy protection, benefit sharing, and ethical oversight rather than consenting to specific studies. Under this model, donated cells are only used in research that adheres to the agreed-upon governance conditions. However, our findings may raise concerns about the applicability of this model to HBO research. While consent for governance could mitigate concerns related to trust, benefit sharing, and potential misuse, it would not fully address the objections of individuals who oppose broad consent due to their desire for study-specific explanations, and their preference for restricting the purpose of the related HBO research. In other words, the consent for governance model does not sufficiently respect the autonomy of individual donors in detail (Lewis and Holm, 2022).

Therefore, if our results are considered sufficiently representative, they suggest that one of the most ethically appropriate method for HBO research in Japan appears to be project-specific consent at least for now, which remains the most obvious way to respect donors' moral views (Greely, 2020; Kataoka et al., 2024a). Of course, project-specific consent increases the burden on researchers in obtaining materials. So, if broad consent is to be maintained, it should, at minimum, be accompanied by a general explanation of the potential use of donated cells in HBO creation (Boers and Bredenoord, 2018). It should be noted here that our data focuses only on specific consent and broad consent, and thus does not address other alternatives that may be ethically desirable (see below). However, regardless of the chosen consent model, ensuring donor autonomy requires that individuals receive adequate explanations about HBO research. This need is particularly urgent given that our findings on public awareness indicate that HBOs are almost entirely unknown to the general public in Japan in the current situation. These claims of ours are broadly consistent with the consensus recently presented by interdisciplinary researchers regarding the provision of cells for brain organoid research: they recommend that, when acquiring new material for HBO research, sufficient explanation about HBOs be provided in the consent form, and that any consent preferences observed be tracked and respected (Cohn et al., 2025). We believe that it is difficult to adhere to these recommendations in broad consent.

We emphasize that the at-least-for-now clause is crucial in our recommendation of specific-consent above. Our results identified various potential reasons against broad consent under the possibility of HBO research. First, there would be individuals who refused broad consent or selected *it depends* due to vague negative feelings or ethical concerns about HBO research in itself. However, at least some of these public concerns could be scientifically unfounded, such as the misconception that an HBO might inherit the donor’s memories or personality (Evans, 2024; Kataoka et al., 2025a). Indeed, among researchers in the field, the prevailing view is that HBO research does not introduce novel ethical concerns that fundamentally differ from those associated with other forms of stem cell research, at least under current and near-future conditions (Hyun et al., 2022). Therefore, science communication can play a critical role in alleviating misconceptions and reducing unnecessary ethical concerns surrounding HBO research. Insights from recent studies examining the psychological factors underlying moral judgements about HBOs may be valuable in shaping public discourse (Evans, 2024; Ota et al., 2024; Villanueva et al., 2025). Moreover, the reasons not being directly related to HBO research are also important. To further enhance public understanding, it is important to address concerns about institutional trustworthiness, research objectives, and potential risks to donors. In addition, both types of reasons for opposition could be amplified by media reports which have often exaggerated ethical concerns surrounding HBO research (Hostiuc et al., 2022; Presley et al., 2022; Kataoka et al., 2023). Mitigating such sensationalism may help influence public attitudes toward HBO research in the future.

That said, it is also crucial to emphasize that even unfounded concerns must be respected in the context of cell donation. Donor autonomy implies that donated cells should not be used in research that donors *perceive* as morally unacceptable. Even if the relevant research is deemed ethically permissible by an ethics committee, for example, the moral values of donors must still be acknowledged and respected. This is particularly important given that public perceptions of HBOs are not entirely “materialistic” in nature unlike the views of most scientists and bioethicists (Evans, 2024).

### 4.3 Limitations

Our claim that HBO research in Japan involves the controversial use of donated cells is, as we have suggested, only tentative due to several limitations. First, while our question emphasized the possibility of cells being used in HBO research, it could not clearly distinguish between those who oppose broad consent because of this very possibility and those who oppose it for more general reasons: among the 36% who oppose broad consent, both groups may be included. Future studies with designs that compare groups that have been exposed to the possibility of HBO research with those that have not will be needed to accurately identify the effects of the possibility of HBO research to the support for broad consent.

Second, since we explicitly stated that we would *not* actually ask for cell donations after the survey, the participants answered the questions in a completely hypothetical manner. This may have prevented us from eliciting participants' genuine attitudes, and they may behave differently if given the actual opportunity.^12^ To address this problem, future survey can incorporate the deception that cell donation opportunities will be provided.

Further, the representativeness of the participants poses certain challenges. The age distribution is skewed toward individuals in their 40s compared to the overall Japanese population, and educational background is biased toward university graduates. Conducting surveys that specifically target younger and older generations would provide a more precise understanding of the distribution of moral views. Additionally, our study was unable to comprehensively explore the depth of each participant’s attitude, highlighting the need for qualitative research to supplement our findings. For instance, as previously noted, some responses about supported research purposes were difficult to interpret logically, suggesting the necessity of a more detailed investigation into which research purposes are deemed acceptable.

Another set of limitations concerns the implications of our results for desirable methods. Mainly due to space limitations, our questionnaire did not examine all candidates for appropriate consent models in full depth.^13^ Therefore, our claim that specific consent is desirable is tentative. For example, in our questionnaire, the explanation of specific consent had to be concise, not fully reflecting its several important elements (such as the need to re-obtain consent for future use). Although people’s attitude of emphasizing the need for detailed explanations has been consistently observed in various surveys, the exact percentages in our results may not be entirely representative.

Even those who did not agree to specific or broad consent might have supported other models. In fact, since our questions primarily focused on the initial consent process, we were unable to directly assess participants’ attitudes toward models emphasizing ongoing engagement in research, whose importance is now strongly emphasized for HBO research (Cohn et al., 2025). One of the most important models we could not handle was dynamic consent: donors can monitor the use of their samples and manage their consent preferences via digital devices (Teare et al., 2021). Although this model needs further consideration in terms of effectiveness and costs, it could help donors avoid involvement in research with controversial use of their donated material. Indeed, previous studies indicate that some individuals expect interim reports on HBO studies to which they have contributed, and prefer greater control over how their donated cells are utilized throughout the research process (Haselager et al., 2020; MacDuffie et al., 2023; Ravn et al., 2023). Future research will need to directly compare various consent methods in the context of HBO research.

### 4.4 Conclusion

The survey reported in this paper, alongside Sawai et al. (2025), represents one of the quantitative studies investigating public attitudes toward HBO research (Evans, 2024; Ota et al., 2024; Villanueva et al., 2025), and the first to specifically examine perspectives on cell donation and consent. Our findings indicate that when participants were informed about the potential creation of HBOs, a substantial proportion of them declined broad consent. Although there are some limitations, this empirical evidence may suggest that HBO research is likely to involve the controversial use of donated cells, at least within the current Japanese context. Therefore, broad consent may not be the most appropriate model for obtaining consent in this research. Preferable alternative would be project-specific consent, while broad consent with general information about HBOs or more dynamic models would be acceptable as well.

While ethical frameworks and regulatory guidelines for biomedical research should ideally aim for universality and international coherence, a culturally sensitive approach is particularly necessary for cell donation, where individual moral beliefs play a central role. Thus, rather than generalizing our findings on cell donation for HBOs beyond the Japanese context, it is crucial to conduct similar social surveys in diverse populations and integrate their results into region-specific consent practices.

Finally, HBO research is not the only domain in which the use of donated biological materials may be ethically contentious. For instance, it may be desirable to inform potential donors that their cells might be used in human-animal chimera research, an area that has raised ethical and societal concerns (Johnston et al., 2022). Public perceptions of what constitutes controversial uses of the donated material are shaped not only by the scientific nature of the research itself but also by citizens' moral frameworks, which in turn are influenced by their knowledge and cultural backgrounds. This underscores the importance of systematically assessing public attitudes through social surveys (Grady et al., 2015) and implementing science communication strategies that can clarify misconceptions and, if appropriate, help shape public understanding.

## Data Availability

The datasets presented in this study can be found in online repositories. The names of the repository/repositories and accession number(s) can be found below: https://figshare.com/articles/dataset/28735757?file=53446964.

## Footnotes

1 The term broad consent is not always unequivocal. On the one hand, in a narrow sense, it can mean approaches that obtain consent for various future research within certain limits of research contents or purposes, falling somewhere between consent for a specific research and completely blanket consent. On the other hand, more broadly, the term can mean any general approach other than specific consent, including blanket consent. In this paper, we use this term in the latter meaning. The discussion below, which is critical of broad consent in the latter, broad sense, can be read as a discussion of the conditions under which broad consent in the former, narrow sense is desirable as well.

2 When brain organoids are created from human embryonic stem cells (hESCs), the donation of embryos is required, and when created from human induced pluripotent stem cells (hiPSCs), the donation of blood is mainly required. In addition, HBOs can also be created from human fetal brain tissues (Hendriks et al., 2024).

3 Here, we distinguish between the possibly controversial nature of HBO research *per se* and the possibly controversial use of cells in HBO research, which is primarily a matter of consent. These two are independent: people may consent to provide cells for research they consider morally problematic since they have other reasons (e.g., altruistic reasons), or they may refuse to provide cells for research they do not consider problematic since, for example, they are simply not interested. Our study focuses solely on citizens' attitudes toward consent; participants' views on HBO research itself are examined elsewhere (Sawai et al., 2025; see also empirical studies cited in the next paragraph).

4 As this study has an exploratory focus, we followed the established guidelines for sample size. Clark and Watson (2016) recommended a minimum sample size of 300 participants ensuring sufficient statistical power. The number of valid responses was determined by excluding those originating from the same connection source within a short interval. As our environment allowed multiple submissions from a single individual, this operation was essential for maintaining data quality.

5 Note that although only four questions are examined in this paper, the remuneration is for responding to the entire questionnaire.

6 Originally recorded in seconds.

7 Further, Akatsuka et al. (2023) has found a similar trend in a survey of Japanese people regarding genome editing on human embryos.

8 Alternatively, the way in which the options were presented may have led to a contrast between basic research and medical applications (we owe this point to a reviewer).

9 Note that the results of the questions about project-specific consent and broad consent cannot be directly compared because the former used a three-point scale while the latter a four-point scale.

10 While some interviewees in MacDuffie et al. (2023) said they would leave it to experts to decide what to do with HBOs because they did not have the expertise, even they wanted to know the results or progress at least.

11 The rate in our results was even lower, which may be interpreted as the effect of priming the possibility of creating HBOs.

12 We owe this point to the reviewers.

13
Lewis and Holm (2024) summarise the advantages and disadvantages of various consent models in the context of HBO research.
