# Supplementary material for: Japanese attitudes toward cell donation in human brain organoid research: many oppose broad consent
Source: Front Genet. 2025 Aug 22;16:1606923. doi: 10.3389/fgene.2025.1606923 (PMC12529070; doi:10.3389/fgene.2025.1606923)
Supplement: Supplementary file 1 [file Supplementaryfile1.docx]

Supplementary Material for

Japanese Attitudes Toward Cell Donation in Human Brain Organoid Research: Many Oppose Broad Consent

Masanori Kataoka, Mayu Koike, and Tsutomu Sawai*

***Correspondence:** [tstmsw@hiroshima-u.ac.jp](mailto:tstmsw@hiroshima-u.ac.jp) (Tsutomu Sawai)

# Supplementary Method

## Note

As explained in the main text, this paper and Sawai et al. (2025) share the same questionnaire and participants, and each report the results of different sections of the questionnaire with different research objectives. For consistency, the following descriptions include the same parts as Sawai et al. (2025) (and its future versions).

The original questionnaire was in Japanese, and the English translation provided below is by the authors. The original version is available on request from the corresponding author.

## Recruitment

We used the Japanese crowdsourcing service Lancers, Inc. (https://www.lancers.jp/) to recruit participants. The survey was posted on the job listing page of the website of Lancers with the title ‘Please cooperate in a questionnaire survey on brain science research’ along with the questionnaire URL. In addition, we used the option to display our project in the PR section of the job listing page and the option to send email notifications to all contractors of Lancers who had received high evaluations from clients in the past, with the aim of quickly recruiting participants and improving the quality of responses.

## Details of the questionnaires related to this study

### *Attention check*

In this section, we presented participants with two questions unrelated to the survey and instructed them on how to respond. Participants could not proceed if they did not provide the specified answers.

[Attention check]

- When answering, it is necessary to read the conditions and contents of the questions carefully. According to past surveys, some participants do not read the questions.
- The following two questions confirm whether you have read the instructions carefully. If you have read this text, please answer the first question with 4 and the second question with half of that number. Only those who answer correctly will be able to proceed to the next question.

Q. I would like to live in a city where I can experience various cultural activities, even if the cost of living is high.

1. Strongly agree
2. Agree
3. Disagree
4. Strongly disagree
5. Neither

Q. I would like to live in a large city rather than a small town.

1. Strongly agree
2. Agree
3. Disagree
4. Strongly disagree
5. Neither

### *Brief overview of brain organoid research*

Considering the intricate nature of brain organoid research, which may be challenging for laypersons to grasp, we provided the following overview of brain organoid research.

[Brief overview]

**What Are Brain Organoids?**

- Significant developments in organoid technology have been made in recent years.
- Organoids are three-dimensional tissues that are created from pluripotent stem cells [1], such as iPS cells [2] or ES cells [3]. The word “organoid” combines the noun “organ,” with the suffix “-oid,” meaning “something like”.
- So far, several different organoids have been grown in the lab, including kidney and liver organoids.
- Using organoid technology, three-dimensional brain tissues were created from human ES cells in 2008, and they were named “brain organoids” in 2013.

[Photos: Brain organoids made from human ES cells, 37 days after creation]^[[1]](#footnote-1)^

Source: Sakaguchi, H., et al. (2019), Self-Organized Synchronous Calcium Transients in a Cultured Human Neural Network Derived from Cerebral Organoids. *Stem Cell Reports* 13(3): 458-473. (partially modified)

[1] Pluripotent stem cells: a cell that has two capacities – (1) the capacity to multiply as much as necessary (self-renewal), and (2) the capacity to become almost any type of cell that constitutes a person (pluripotency).

[2] iPS cell: a type of pluripotent stem cell that is created by introducing specific genes into body cells, such as skin cells and blood cells.

[3] ES cell: a type of pluripotent stem cell that is created from cells extracted from a fertilized egg (embryo) several days after fertilization.

**Various Research Purposes**

- Human brain organoids are produced in the lab by mimicking the early process of brain formation inside the mother's womb. Therefore, brain organoids are expected to be used for the following purposes:

1. Basic research into understanding how the brain is formed.
2. Basic research into understanding why brain-related diseases occur.
3. Applied research to identify effective drugs for brain-related diseases.
4. Applied research to develop effective treatments for brain-related diseases.
5. Medical application of transplanting human brain organoids to treat the loss of (human) brain functions due to car accidents or strokes and so forth.

**Ethical Issues**

- Various ethical issues have already been raised in relation to brain organoid research. Here are some of them:

1. Complex brain activity has been detected in human brain organoids. This may mean that human brain organoids are already conscious.
2. Human brain organoids that are connected to retinal cells respond to light. This may mean that human brain organoids can already “see” light.
3. Research is underway to transplant human brain organoids into the brains of animals such as rats and monkeys. This could result in these animals acquiring advanced cognitive abilities more similar to those of humans.

**The Current Status and Future of Brain Organoid Research**

- The brain organoids that are being produced today are highly immature compared to human brains, in terms of their structure, size, and maturity. They also lack the input systems (nose, mouth, and ears) and other related systems that enable us to obtain information from the outside world, and they are therefore thought to be incapable of fully developing senses, thoughts and emotions.
- However, it has been suggested that more sophisticated human brain organoids could be produced in the future, in which case the various ethical issues that are currently of concern could become reality.

### *Comprehension test*

The comprehension test used the following question: “For each of the following statements about organoids, please select the one option that comes closest to your understanding”. The response options were: “Definitely wrong”; “Probably wrong”: “Probably right”; “Definitely right”; and “Do not know”. Information on statements was provided to the participants in the brief overview described above. Participants were presented with the following 10 statements.

[Comprehension test]

1. “It is now possible to produce three-dimensional tissues, called organoids, outside the body.” (R)
2. “By organoid technology, brain parts are now being created, but not other body parts.” (W)
3. “Pluripotent stem cells – such as iPS cells and ES cells – are needed to produce organoids.” (R)
4. “Human brain organoids are used to understand how our brains are formed, but not to create drugs.” (W)
5. “Human brain organoids could be used in the future to treat strokes, etc.” (R)
6. “There are no ethical issues relating to the creation and use of human brain organoids.” (W)
7. “No ethical concerns have arisen in relation to the research transplanting human brain organoids into animal brains.” (W)
8. “Human brain organoids have a structure as complex as our human brains.” (W)
9. “Human brain organoids already have senses, thoughts and emotions.” (W)
10. “No matter how advanced the technology could become in the future, it would be impossible to create a brain organoid with the same structure as the human brain.” (W)

Each statement was categorized as either right (R) or wrong (W). For R items, the correct responses were “Definitely right” or “Probably right”; for W items, they were “Definitely wrong” or “Probably wrong.” A score of 1 was assigned for each correct response, and 0 for all others. Therefore, the highest score on the comprehension test was 10. The percentages of correct answers for each question were as follows: Q1, 94%; Q2, 63%; Q3, 90%; Q4, 74%; Q5, 90%; Q6, 87%; Q7, 87%; Q8, 52%, Q9, 65%; and Q10, 68%. Note that the test score is not used in the analysis of this study.

### *Awareness-related question regarding brain organoids*

In this section, we asked whether participants were aware of brain organoids by asking the following question.

[Awareness]

Q. Before participating in this survey, were you aware of brain organoids?

- I knew enough about brain organoids to be able to explain them to some extent.
- I had heard of brain organoids, but did not know much about them.
- I did not know about brain organoids.

### *Questions about cell donation for brain organoid research*

This section begins by giving participants a brief explanation of cell donation for human brain organoid research, then presents three questions. Horizontal lines indicate page breaks.

[Explanation]

Below is an explanation of cell donation. Please read the explanation and select the option that best fits your thoughts on the questions that follow.

- Human iPS cells are one of the cell types needed to create human brain organoids.
- iPS cells are primarily derived from blood cells, meaning that blood donation is required to generate human brain organoids.
- Cell donors are typically provided with a detailed explanation of the research and its associated risks. Only after fully understanding these details do they decide whether to consent to donating their cells for that research.

[Willingness]

Q. Would you be willing to donate your cells to create iPS cells for human brain organoid research?

*You will **NOT** be asked to donate your cells as part of this study.

1. I would be willing to donate my cells.
2. It would depend on the purpose of the study.
3. I would not be willing to donate my cells.

[Purposes]

Q. For which of the following purposes would you be willing to donate your cells? (You can select multiple answers)

1. To deepen our understanding of the brain (for example, brain development, structure, and function)
2. To elucidate the causes of brain-related diseases
3. To develop treatments for brain-related diseases
4. To develop drugs for brain-related diseases
5. To elucidate the causes of brain-related diseases of yours, or to develop treatments and drugs for them
6. To elucidate the causes of brain-related diseases in your family, or to develop treatments and drugs for them
7. I would not be willing to donate my cells for any of the purposes above.

Are there any purposes for which you would **NOT** be willing to donate your cells? (within 1000 characters)

*If nothing, please write “nothing”.

[Broad consent]

At some research institutions, researchers have the authority to determine how donated cells will be used. Therefore, if you were to donate your cells to such an institution, brain organoids could be produced from your donated cells.

Q. Would you be willing to donate your cells to such an institution?

1. I would be willing to donate my cells.
2. I would not be willing to donate my cells.
3. I don’t know.
4. It would depend on the purpose of the study.

Please describe why you think that way (within 1000 characters).

*If no reason in particular, please write “nothing”.

## References

- Sawai, T., Koike, M., Kataoka, M. (2025). Human brain organoid research: An analysis of public attitudes and ethical concerns in Japan [version 1; peer review: 2 approved with reservations, 1 not approved]. Mol. Psychol. 4, 1. doi: 10.12688/molpsychol.17590.1.

# Supplementary Table

## Supplementary Table 1. Demographic information of the participants.

| **Demographic information** | **N** |
| --- | --- |
| ***Total*** | 326 |
| ***Country*** |  |
| Japan | 326 |
| ***Age (in years)*** |  |
| ≤19 | 1 |
| 20­­–29 | 23 |
| 30­–39 | 99 |
| 40–49 | 138 |
| 50­–59 | 50 |
| 60­–69 | 15 |
| ≥70 | 0 |
| ***Gender*** |  |
| Female | 126 |
| Male | 200 |
| Other | 0 |
| Declined to state | 0 |
| ***Education*** |  |
| Elementary School | 0 |
| Junior High School | 7 |
| High School or Technical College | 66 |
| Vocational School | 36 |
| Junior College | 23 |
| Bachelor’s Degree | 179 |
| Master’s Degree | 13 |
| Ph.D. | 2 |
| Other | 0 |
| ***Brain-related diseases*** |  |
| Yes | 5 |
| No | 315 |
| Declined to state | 6 |
| ***Brain-related diseases (family)*** |  |
| Yes | 21 |
| No | 301 |
| Declined to state | 4 |
| ***Companion animal*** |  |
| Yes | 92 |
| No | 231 |
| Declined to state | 3 |
| ***Religions*** |  |
| Yes | 39 |
| Christianity | 9 |
| Buddhism | 22 |
| Islam | 0 |
| Shinto | 5 |
| Hinduism | 0 |
| Other | 2 |
| Declined to state | 1 |
| No | 281 |
| Declined to state | 6 |

1. Photos are omitted. [↑](#footnote-ref-1)
